# From where did the Western honeybee (*Apis mellifera)* originate?

**DOI:** 10.1002/ece3.312

**Published:** 2012-07-12

**Authors:** Fan Han, Andreas Wallberg, Matthew T Webster

**Affiliations:** Science for Life Laboratory, Department of Medical Biochemistry and Microbiology, Uppsala UniversitySweden

**Keywords:** Bioinformatics, genomics, population genetics

## Abstract

The native range of the honeybee *Apis mellifera* encompasses Europe, Africa, and the Middle East, whereas the nine other species of Apis are found exclusively in Asia. It is therefore commonly assumed that *A. mellifera* arose in Asia and expanded into Europe and Africa. However, other hypotheses for the origin of *A. mellifera* have also been proposed based on phylogenetic trees constructed from genetic markers. In particular, an analysis based on >1000 single-nucleotide polymorphism markers placed the root of the tree of *A. mellifera* subspecies among samples from Africa, suggestive of an out-of-Africa expansion. Here, we re-evaluate the evidence for this and other hypotheses by testing the robustness of the tree topology to different tree-building methods and by removing specimens with a potentially hybrid background. These analyses do not unequivocally place the root of the tree of *A. mellifera* subspecies within Africa, and are potentially consistent with a variety of hypotheses for honeybee evolution, including an expansion out of Asia. Our analyses also support high divergence between western and eastern European populations of *A. mellifera*, suggesting they are likely derived from two distinct colonization routes, although the sources of these expansions are still unclear.

## Introduction

**Figure 1:**
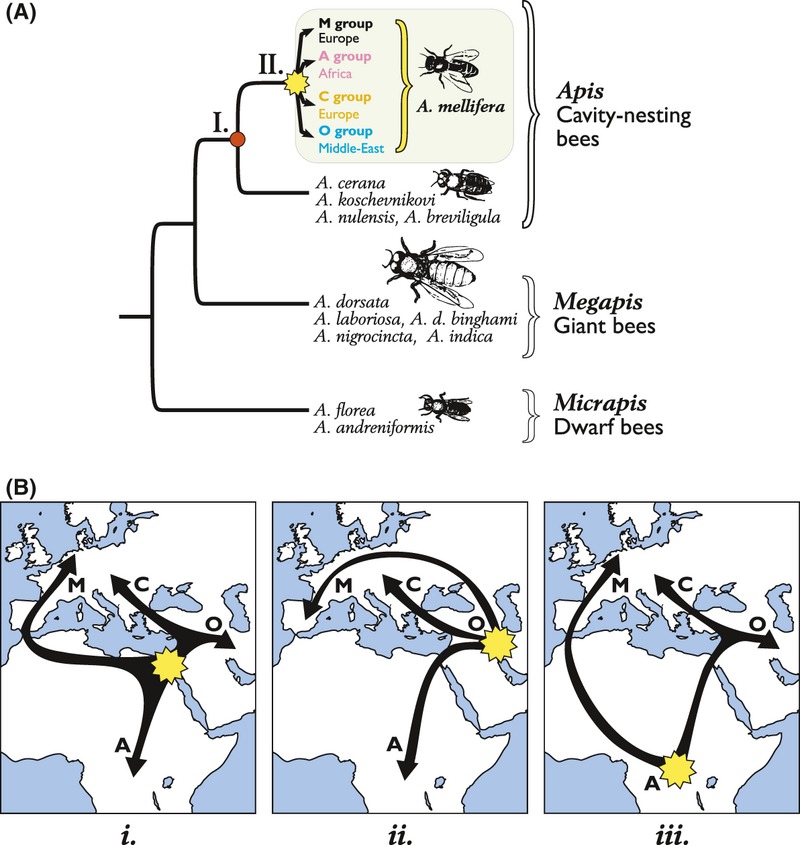
Evolution of *Apis mellifera*. (A) Phylogeny representing the three clades of Apis. All of the 10 extant Apis species apart from *A. mellifera* are found only in Asia. Node I represents the split between *A. mellifera* and other cavity-nesting bees. Node II represents the most recent common ancestor of extant subspecies of *A. mellifera*. (B) Three hypotheses that have been proposed for the origin of *A. mellifera*. (i) An expansion from the Middle East, involving colonization of Europe via two routes, one eastern and one western was first suggested by Ruttner (1978) on the basis of morphometric analyses. (ii) An expansion from the Middle East, which did not involve the western colonization route into Europe was suggested on the basis of trees constructed from mtDNA (Garnery et al. 1992). (iii) An origin in Africa was proposed by Wilson (1971) and an expansion out of Africa via both an eastern and western route was suggested by the analysis of >1000 SNPs by Whitfield et al. (2006). The yellow star corresponds to node II in the upper panel.

The Western honeybee, *Apis mellifera*, is a species of crucial economic, agricultural, and environmental importance. Due to the activities of beekeepers it is now spread across the entire world, but its native range is large and diverse, spanning Europe, Africa, and the Middle East. Including *A. mellifera*, 10 species of honeybee belonging to the genus Apis are generally recognized (Engel 1999; Arias and Sheppard 2005). Phylogenetic analyses based on nuclear DNA and mitochondrial (mtDNA) markers strongly support clustering these into three distinct groups: cavity-nesting bees (*A. mellifera, A. cerana, A. koschevnikovi, A. nulensis*), giant bees (*A. dorsata, A. laboriosa, A. binghami, A. nigrocincta*), and dwarf bees (*A. florea, A. andreniformis*) (Arias and Sheppard 2005; Raffiudin and Crozier 2007) (Fig. 1A). Apart from *A. mellifera* all of these species are currently confined to Asia and the lineage that gave rise to extant *A. mellifera* represents an early split from other cavity-nesting bees, so it is most likely that *A. mellifera* can ultimately trace its origin to Asia.

At least 29 subspecies of *A. mellifera* have been delineated on the basis of morphometry (Ruttner 1988; Engel 1999; Sheppard et al. 2003). These subspecies are now typically divided into four major groupings, supported by morphometric and genetic studies in addition to analyses of ecological, physiological, and behavioral traits: group A, which includes subspecies throughout Africa; group M, which includes subspecies from western and northern Europe; group C, which includes subspecies from eastern Europe; and group O, which includes species from Turkey and the Middle East (Ruttner et al. 1978; Ruttner 1988; Garnery et al. 1992; Arias and Sheppard 1996; Franck et al. 2000; Miguel et al. 2011). However, some studies do not distinguish between groups C and O (labeling them both as C) (Ruttner 1988; Cornuet and Garnery 1991; Garnery et al. 1992) and the existence of a fifth lineage (Y) in north-east Africa has been proposed (Franck et al. 2001).

The *A. mellifera* lineage split from other cavity-nesting bees, and eventually diversified into subspecies and colonized their present native range. Estimates from genetic divergence of mtDNA and nuclear loci suggest that the first split occurred between 6 and 9 million years ago (Cornuet and Garnery 1991; Arias and Sheppard 2005). In contrast, genetic variation between the extant subspecies of *A. mellifera* is mostly shared, which suggests they have not experienced long periods of isolation. Genetic dating of mtDNA lineages suggests that the four major subspecies groups diverged around 0.7–1.3 million years ago (Garnery et al. 1992; Arias and Sheppard 1996). There is little data available to infer the geographical ranges that *A. mellifera* inhabited in the time between the split from the ancestors of other cavity-nesting bees (more than 6 million years ago) and their colonization of their present ranges (beginning around 1 million years ago). However, genetic and morphological relationships between extant subspecies can be used to infer the timing and location of their common origin.

Three main scenarios have been proposed for the evolutionary origin of *A. mellifera*, summarized in Figure 1B. The first, initially proposed by Ruttner et al. (1978) suggests that *A. mellifera* has its historic center in the Middle East or northeast Africa from where it colonized Europe through two routes: a direct eastern route and a western route via north Africa and the Iberian peninsula (Fig. 1B, i). This hypothesis was based on morphological analysis that suggests continuity between the A (Africa) and M (W and N Europe) lineages and an ancestral form close to *A. m. syriaca* from Lebanon, Israel, and Jordan. Another hypothesis, based mainly on mtDNA analyses (Cornuet and Garnery 1991; Garnery et al. 1992) also proposes a Middle Eastern origin, but does not include colonization of Europe via a western route (Fig. 1B, ii). This scenario is based on a phylogenetic tree that groups the A lineage with C rather than M, arguing against migration across the strait of Gibraltar.

The hypothesis of an African origin was espoused by E. O. Wilson (quoting C. D. Michener) based on an assumption that the ability of domestic *A. mellifera* to form a winter cluster represents a derived adaptation to temperate climates (Wilson 1971). It was argued that because *A. mellifera* does not presently occur in tropical Asia, an African origin of the hypothesized ancestral tropical form was more likely. The most comprehensive genetic study to date, based on 1136 nuclear single-nucleotide polymorphisms (SNPs) is typed in 341 individuals from 14 geographical subspecies of *A. mellifera* and three outgroups (*A. cerana*, *A. florea*, *A. dorsata*) argued in favor of this hypothesis (Whitfield et al. 2006). This analysis supported the classifications into four lineages (A, C, M, O) based on previous morphological and genetic analyses. The M (W Europe) and C (E Europe) lineages were found to be highly divergent, with the M lineage grouping with A (Africa) and the C lineage with O (Middle East). The outgroups were used to root the tree, which occurred within the A lineage, separating *A. m. intermiss*a (from the extreme north-west of Africa) from the other members of the A group. The tree therefore splits into two main clades, one grouping the M lineage *(A. m. mellifera* and *A. m. iberiensis*) together with *A. m. intermiss*a and the other containing all other subspecies of the A, C, and O lineages. Based on the position of the root within the A lineage, it was suggested that modern populations of *A. mellifer*a can trace their origin to Africa via two distinct migrations – a western expansion of the M lineage into Europe and one or more eastern expansions of the O and C lineages into Europe and Asia (Fig. 1B, iii).

Understanding the origin of *A. mellifera* is important for tracing the evolution of novel and local adaptations in a species increasingly threatened by disease, climate change, habitat loss, and introgression (De la Rúa et al. 2009; Potts et al. 2010). Large differences in physiology and behavior occur between African and non-African subspecies (Ruttner 1988; Hepburn and Radloff 1998). In general, African subspecies exhibit migratory behaviors, high reproduction rates, and strong defensive behavior, whereas in contrast, subspecies in temperate climates are more stationary, with a lower reproduction rate, and less aggressive defense. An important adaptation to temperate climates is the ability to form a winter cluster and survive without flying for at least 5 months of the year. Furthermore, different subspecies differ in disease resistance. Understanding their evolutionary past could be the key to understand how these adaptations arose. Did newly acquired adaptations to cold enable *A. mellifera* to colonize Europe from Africa, or did the source population already posses such adaptations?

Here, we reanalyze data presented in Whitfield et al. (2006) and review previous genetic analyses to evaluate support for the various hypotheses for the origin of *A. mellifera*. We first assay levels of unique and shared variation among *A. mellifera* lineages and subspecies. A large proportion of genetic variation is shared between *A. mellifera* subspecies, which suggests that they have not experienced long periods of isolation and substantial gene flow is likely making interpretation of bifurcating trees problematic. We perform analyses using different measures of genetic distance and tree construction and explore the robustness of the tree topology to remove subspecies, particularly *A. m. intermissa*, which appears to have unclear ancestry. We use these analyses to assay evidence for an African or Asian origin of *A. mellifera*, and for the existence of a colonization route into Europe via Africa through the Iberian Peninsula.

## Materials and Methods

### Samples

Detailed information about samples, locations, and SNPs identification are described in Whitfield et al. (2006). The data set consisted of 35 samples of *A. mellifera* subspecies from East Europe (17 *A. m. carnica*, 18 *A. m. ligustica*), 31 from West Europe (20 *A. m. mellifera*, 11 *A. m. iberiensis*), 42 from Asia (19 *A. m. anatoliaca*, 11 *A. m. caucasica*, 9 *A. m. syriaca*, 3 *A. m. pomonella*), and 67 from Africa (19 *A. m. intermissa*, 22 *A. m. scutellata*, 19 *A. m. lamarckii*, 3 *A. m. capensis*, 2 *A. m. litorea*, 2 *A. m. unicolor*). Samples from three related Apis species were also included (7 *A. cerana*, 2 *A. florea*, 4 *A. dorsata*).

### Differentiation estimates and phylogenetic analysis

Levels of genetic variation within each subspecies corrected for sample size were estimated by calculating Watterson's *θ* (Watterson 1975) using a custom perl script. In order to analyze the relationships between the 14 subspecies of *A. mellifera*, we measured the degree of genetic differentiation between each pair of populations by estimating *F*_ST_ (Fixation index), including the three outgroup species using a custom perl script (Weir and Cockerham 1984). In addition, we also directly inferred the pairwise genetic distances among individual bee samples from the SNP data using allele-sharing distance in *plink* (Purcell et al. 2007). Phylogenetic trees were generated using the neighbor-joining algorithm in *phylip* (Saitou and Nei 1987). The neighbor-joining trees were plotted with *SplitsTree* (Huson and Bryant 2006). This program was also used to construct networks from the distance matrices based on both allele-sharing and *F*_ST_-based distances. The SNP data set was randomly resampled a hundred times to generate a set of bootstrap replicates from which new distance matrices and a majority-rule consensus tree were computed. To illustrate the alternative positions of the outgroup in the absence of the *A. m. intermissa* subspecies, PAUP* (Swofford 2003) was used to filter out trees compatible with the majority-rule solution and to compute a second consensus from the remaining trees.

## Results

**Figure 2:**
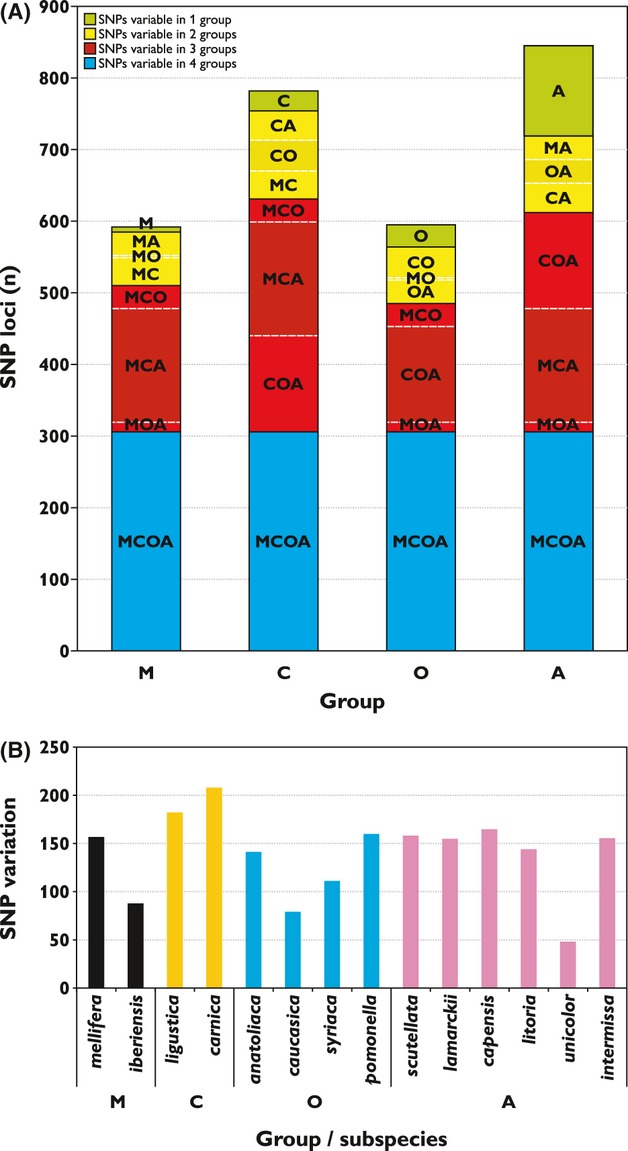
Patterns of SNP among subspecies of *Apis mellifera*. (A) Total numbers of SNPs identified in the four groups of *A. mellifera* subspecies. Each SNP is categorized by the set of groups in which it is observed to be polymorphic. The majority of SNPs are variable in at least three groups, and very few are restricted to a single group. (B) Levels of genetic variation among *A. mellifera* subspecies estimated using Watterson's estimator (Watterson 1975). Subspecies are grouped according to lineage group (M, C, O, and A).

In total, 1029 SNPs from Whitfield et al. (2006) exhibit variation within and/or between *A. mellifera* subspecies (Table 1 and Fig. 2A). The majority of SNPs are variable in multiple subspecies and across multiple groups, suggesting that genetic variation is mainly shared across the entire range of subspecies. An average of 414 (40%) SNPs are polymorphic in any one subspecies in this data set and an average of 703 (68%) SNPs are polymorphic in any one group. Three hundred and six (30%) SNPs are polymorphic in all four groups. By contrast, only an average of five (0.5%) SNPs are unique to a subspecies and an average of 47 (4.5%) SNPs are found only in one group. There is only one example of a SNP that is fixed in a subspecies (in *A. m. unicolor*) but not present in other subspecies, and no examples of SNPs that are fixed in a group but absent in other groups. That the majority of genetic variation is shared indicates that *A. mellifera* subspecies have not experienced long periods of isolation and makes interpretation of bifurcating trees problematic.

**Table 1:** Number of SNP loci that are polymorphic in samples of each subspecies of *Apis mellifera*

Lineage	Subspecies	Number of samples	Polymorphic SNPs	Private SNPs	Fixed private SNPs
M	*mellifera*	20	564	4	0
	*iberiensis*	11	265	2	0
	All group	31	592	7	0
C	*ligustica*	18	637	8	0
	*carnica*	17	715	4	0
	All group	35	782	24	0
O	*anatoliaca*	19	501	2	0
	*caucasica*	11	239	0	0
	*syriaca*	9	314	4	0
	*pomonella*	3	293	0	0
	All group	42	595	31	0
A	*scutellata*	22	584	34	0
	*lamarckii*	19	549	3	0
	*capensis*	3	302	3	0
	*litorea*	2	216	1	0
	*unicolor*	2	72	1	1
	*intermissa*	19	552	5	0
	All group	67	845	126	1
Total	All groups	175	1029	–	–

Figure 2B shows levels of genetic variation within each subspecies corrected for sample size (Watterson 1975). Although there is some variation between subspecies, many of the subspecies with unusual levels of variation have small sample size, and thus measures are less reliable. Levels of variation among groups do not differ substantially, and therefore there is no support for a greater genetic diversity in Africa, which has been found in previous studies (Estoup et al. 1995; Franck et al. 2001). The SNP ascertainment biases must be considered in such an analysis. In this study, ascertainment of SNPs came from two sources: alignment of genome traces of Africanized honeybees (with European and African ancestry) to the genome assembly (European-derived) (54% of polymorphic SNPs) and alignments of ESTs derived from European honeybees to the genome assembly (46% of polymorphic SNPs). Hence, this ascertainment scheme is expected to mainly identify SNPs that are variable within Europe, with a smaller contribution from SNPs variable between Europe and Africa. However, as we have observed that SNPs are mostly shared between subspecies and groups, the ascertainment scheme is unlikely to cause large biases in estimations of levels of variation between subspecies.

**Figure 3:**
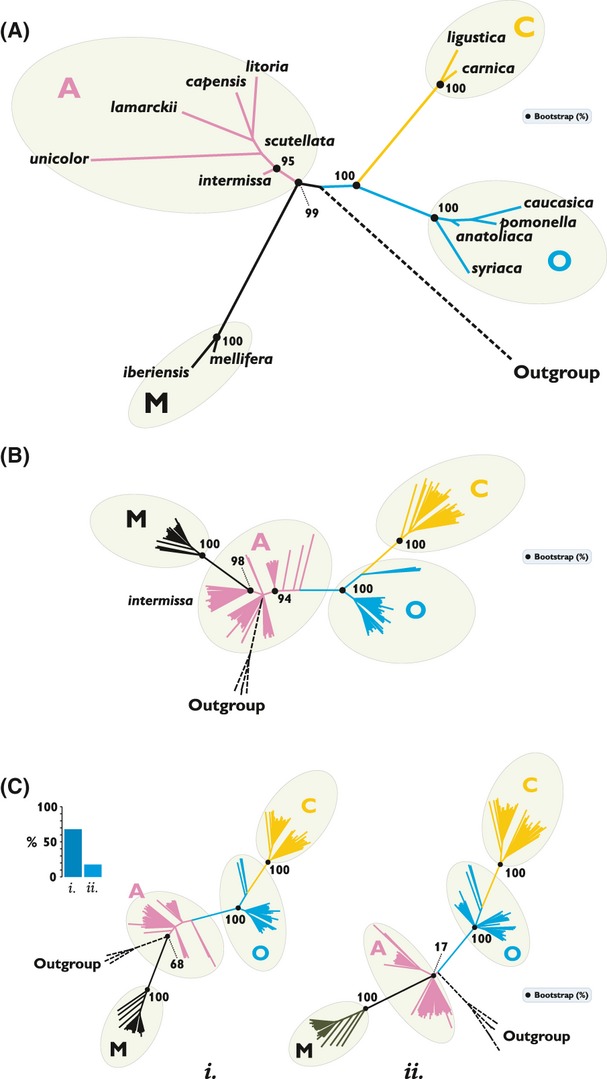
Trees representing the relationship between subspecies of *Apis mellifera* based on SNP data presented in Whitfield et al. (2006). (A) Consensus neighbor-joining tree based on pairwise *F*_ST_ distances between the subspecies and three outgroups combined. Subspecies cluster into four previously defined groups (M, C, O, and A), and the root branches from the tree between the A+M and C+O clusters. (B) Neighbor-joining majority-rule consensus tree based on allele-sharing distances among all subspecies and outgroup samples derived from 100 bootstrap replicates. As observed by Whitfield et al. (2006), the outgroup is firmly attached among African subspecies. (C) Two competing reconstructions with regards to position of the outgroup are recovered when the subspecies *A. m. intermissa* is removed from the data set: (i) The outgroup clusters with the M group in 68% of the bootstrap replicates. (ii) In the second most strongly supported position (17% of the trees), the outgroup is instead positioned between the A+M and C+O groups. The diagram shows the relative weight of the two reconstructions.

We investigated the relationship between subspecies by inferring neighbor-joining trees based on *F*_ST_ distances among the 14 *A. mellifera* subspecies and three outgroup species (Fig. 3A). The clustering of subspecies strongly supports previous analyses based on morphometric and genetic data, with four distinct groups corresponding to the A, M, C, and O lineages. Furthermore, the two European groups (M and C) are distantly related, clustering with the A and O groups, respectively, as in previous analyses. Importantly, the shape of the *F*_ST_ trees differs from that in Whitfield et al. (2006), which was based on allele-sharing distances between individual samples (reproduced in Fig. 3B): the root of the *F*_ST_ tree separates the A+M from the C+O clusters, rather than falling within Africa as indicated by the allele-sharing tree. Instead of the A-group appearing as a paraphyletic assemblage giving rise to the European M lineage and the C+O lineages and with *A. m. intermissa* holding an intermediate position between the A and M groups, the African subspecies form a monophyletic clade.

The previous analyses by Whitfield et al. (2006) using structure clearly delineated nearly all subspecies and clustered them into previously defined groups. However, one subspecies, *A. m. intermissa*, stands out as a mixture between A and M ancestry (Whitfield et al. 2006, p. 642; Fig. 1C). This may indicate that this subspecies has a recent hybrid origin, or that the particular samples were not true representatives of the subspecies. Furthermore, the *A. m. intermissa* samples branch closer to the M group than do the other African subspecies, which may indicate they contain a mixture of A and M group ancestry, as indicated by the structure analysis (Whitfield et al. 2006). We thus decided to reconstruct phylogenetic networks as well as performing further analyses of allele sharing while removing *A. m. intermissa* to ascertain the robustness of the neighbor-joining trees when facing conflicting genealogical signals potentially due to hybridization.

**Figure 4:**
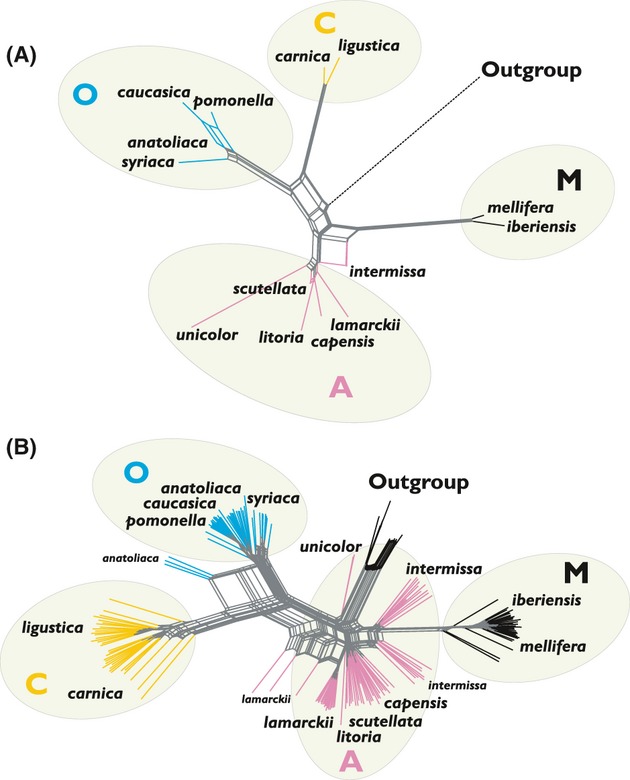
Phylogenetic networks illustrative of the ambiguity regarding the position of the outgroup. (A) Network constructed from *F*_ST_ distances between the lineage groups. (B) Network constructed from allele-sharing distances between samples.

When *A. m. intermissa* is included in the analysis, the outgroup is attached with high-bootstrap support among other African subspecies (Fig. 3B). However, when *A. m. intermissa* is removed we find that the bootstrap replicates recover two main competing positions for the outgroup, neither of which firmly and unequivocally places it among those African subspecies (Fig. 3C). Instead, the outgroup either clusters with the M group (68% of the bootstrap trees), or at the branch separating A+M from C+O (17% of the trees), the latter of which closely matches the *F*_ST_-based tree (Fig. 3A). Hence, we find that the position of the root in Africa is heavily affected by the *A. m. intermissa* samples and not decisively supported by the rest of the data. However, the data do not decisively support a single topology for the relationship between lineage groups. In order to visualize conflicts in the data we used networks. A network visualizing the *F*_ST_ distance matrix reveals a few interesting features (Fig. 4A). First, *A. m. intermissa* occupy a position intermediate to A and M as suggested by previous analyses. Second, there are incongruencies in the center of the tree indicating that multiple topologies for the relationship between the four groups and the root are supported by the data. A similar picture is shown by considering each sample individually (Fig. 4B; shown in more detail in Fig. S1). In this analysis, the root branches close to the A group, although not unequivocally within it. Interestingly, a number of other samples with potentially hybrid origin can be identified in this tree. Some samples of *A. m. anatoliaca* (O group) and *A. m. lamarckii* (A group) appear closer to the C group. This could potentially indicate recent hybridization due to beekeeping with a popular race such as *A. m. carnica* (C group). In addition, samples of *A. m. intermissa* appear intermediate to the A and M groups, as previously observed. It is clear from these analyses that samples cluster tightly into the four lineage groups, but that within each group, subspecies are less easily distinguishable from each other by genetic differences.

A link between the A and M clusters is supported by morphological analysis and by the analysis of Whitfield et al. (2006), although not by some other genetic studies (Cornuet and Garnery 1991; Garnery et al. 1992). Such a link could represent the signal of an ancient colonization of western and northern Europe via northwest Africa. However, more recent hybridization could also generate this pattern. We constructed an additional tree with both *A. m. intermissa* and *A. m. iberiensis* removed, as these subspecies occur close to the boundary between Western Europe and North Africa (Fig. S2). We found the topology consistent with those presented in Figure 2C, where the closest relatives of the M group are within the A group. This indicates a closer relationship between western European and sub-Saharan African honeybees than between western and eastern European honeybees. There is therefore good evidence for a link between the A and M groups.

## Discussion

The major evolutionary events that have determined the current genetic structure of modern *A. mellifera* are (1) an ancient split with other cavity-nesting honeybee species followed by (2) dispersal and differentiation of subspecies across their native ranges of Europe, Africa, and the Middle East, and finally (3) further dispersal and admixture of subspecies via the activities of humans. The oldest evidence for an association between humans and honeybees is a cave painting in Spain depicting honey hunting, dated at around 7000 years ago (Crane 1999). However, there is little archaeological evidence for the timing of the earlier events. The oldest fossil remains of honeybees belong to the Oligocene, but no honeybee fossils have been found from the Pliocene, the period when, according to molecular dating, *A. mellifera* had already diverged from their closest extant relatives (Garnery et al. 1992; Kotthoff et al. 2011). Based on comparisons of mtDNA loci, the *A. mellifera* lineage split from other extant honeybees at least 6 million years ago, and the subspecies began diverging around 1 million years ago. These estimates are based on a commonly used rate of mtDNA sequence divergence in insects of 2% per million years (Brower 1994). The reliability of this estimate is not certain (Papadopoulou et al. 2010) and in the absence of recent fossils it is not possible to refine specifically for honeybee clades. It is, however, most likely that the divergence of honeybee subspecies is due to ancient migrations rather than more recent events such as the expansion out of Pleistocene glacial refugia, or even more recent dispersal with humans.

It seems reasonable to assume that the original emergence of the *A. mellifera* lineage occurred in Asia, where the other nine honeybee species are currently found. The source of modern populations of *A. mellifera* is, however, unclear. There are two main points of disagreement between hypotheses for their origin. First, was the source population found in Africa or the Middle East? Second, did colonization of Europe include a western route via northwest Africa through the Iberian Peninsula? The following combinations of these features have been proposed: (a) a Middle-Eastern origin, including colonization of Europe via a western route (Ruttner et al. 1978; Ruttner 1988) (Fig. 1B, i), (b) a Middle-Eastern origin, not including the western colonization route (Garnery et al. 1992) (Fig. 1B, ii), and (c) an African origin, including the western colonization route (Whitfield et al. 2006) (Fig. 1B, iii).

The presence of at least four distinct lineage groups of *A. mellifera* (A, C, O, M) subspecies is well supported by genetic and morphological evidence. However, studies have come to conflicting results about how these clusters are related. One point of contention is whether there is evidence for a close relationship between the A and M lineages, which could support a western route of colonization of Europe. Such a link is indicated from the morphological evidence (Ruttner et al. 1978; Ruttner 1988). However, studies based on mtDNA do not cluster these groups together (Cornuet and Garnery 1991; Garnery et al. 1992) instead favoring a clustering of the C and A groups. This topology has very low bootstrap support, however, suggesting that while major lineage groups are well supported, relationships between them are more problematic to distinguish. Several studies based on microsatellite and mtDNA markers demonstrate that subspecies from the A and M lineage groups are clearly distinguishable, although many haplotypes are shared (Franck et al. 1998; De la Rua et al. 2002; Cánovas et al. 2007; Miguel et al. 2011). Analysis of the present SNP data set suggests that the M group is most closely related to A, and most distantly related to C, which is also found in Europe. This provides good evidence that the two European lineages are independently derived, and supports the existence of a western route of migration into Europe from Africa.

The other point of discussion is where modern subspecies of *A. mellifera* originated. One question is whether the source population of expansions of *A. mellifera* was adapted to temperate climates. One theory maintains that development of these adaptations allowed expansion into Europe from Africa, where the only native tropical populations of *A. mellifera* are found today (Wilson 1971). However, modern populations of *A. cerana* inhabit temperate climates within Asia (Corlett 2011), so cold tolerance could be an ancestral adaptation of cavity-nesting honeybees. There is also evidence that genetic variation in microsatellite loci in African subspecies is higher than other lineage groups (Estoup et al. 1995; Franck et al. 2001). Higher African diversity is not observed in the present SNP data set, although as this does not represent an unbiased sample of SNPs and additional diversity in African subspecies may be missed. It is plausible that effective population sizes are larger in Africa. Presently, wild colonies of *A. mellifera* in Africa are numerous, whereas in Europe honeybees are mainly restricted to managed colonies at a much lower density (Moritz et al. 2007; Dietemann et al. 2009). Furthermore, African subspecies are unlikely to have suffered population bottlenecks due to quaternary ice ages to the same extent as European populations. Higher genetic diversity in Africa need not therefore indicate an out-of-Africa expansion and could simply reflect a larger long-term effective population size.

Insight into the origin of *A. mellifera* can also be gained from locating the position of the root of the tree of subspecies. This is problematic because of the large amount of shared variation and inconsistencies in internal branches. The root appears close to the node joining A and M branches but different analyses place it either within A lineages, or ancestral to both lineage groups. The SNP data therefore does not provide an unequivocal answer to the root of the tree. Here, we have shown that the shape of the SNP tree of *A. mellifera* subspecies is sensitive to inclusion of *A. m. intermissa*, which does not appear genetically distinct. The *A. m. intermissa* samples included in this data set could have been affected by gene flow from the M lineage group, or alternatively the results could indicate a hybrid origin of *A. m. intermissa*. It is likely that unbiased sampling of SNPs will provide a more comprehensive answer. In summary, while it is not possible to conclusively rule out any of the major hypotheses for the origin of *A. mellifera*, the hypothesis first proposed by Ruttner (1978) (Fig. 1B, i) involving an expansion from an area close to where other Apis species are presently found, fits well with current evidence.

A full understanding of origin of *A. mellifera* is important for several reasons. During colonization of current ranges they experienced strong selection for adaptation (Zayed and Whitfield 2008). A correct understanding of the origin and routes by which *A. mellifera* colonized new environments is crucial for understanding how and when these adaptations arose. Furthermore, subspecies differ in their susceptibility to major diseases and African subspecies in particular appear to have greater tolerance to *Varroa destructor* mites (Dietemann et al. 2009). Which adaptations are derived and which were already present in the ancestral populations? The answer to these questions is important for our understanding of honeybee biology and could have implications for their conservation.

## Supporting Information

**Figure S1.** Network constructed from allele-sharing distances between samples including individual sample labels.

**Figure S2.** Neighbor-joining tree constructed from allele-sharing distances between samples excluding *A. m. intermissa* and *A. m. iberiensis*.
