# Supplementary figures and images for: From where did the Western honeybee (Apis mellifera) originate?
Source: Ecol Evol. 2012 Jul 12;2(8):1949–57. doi: 10.1002/ece3.312 (PMC3433997; doi:10.1002/ece3.312)

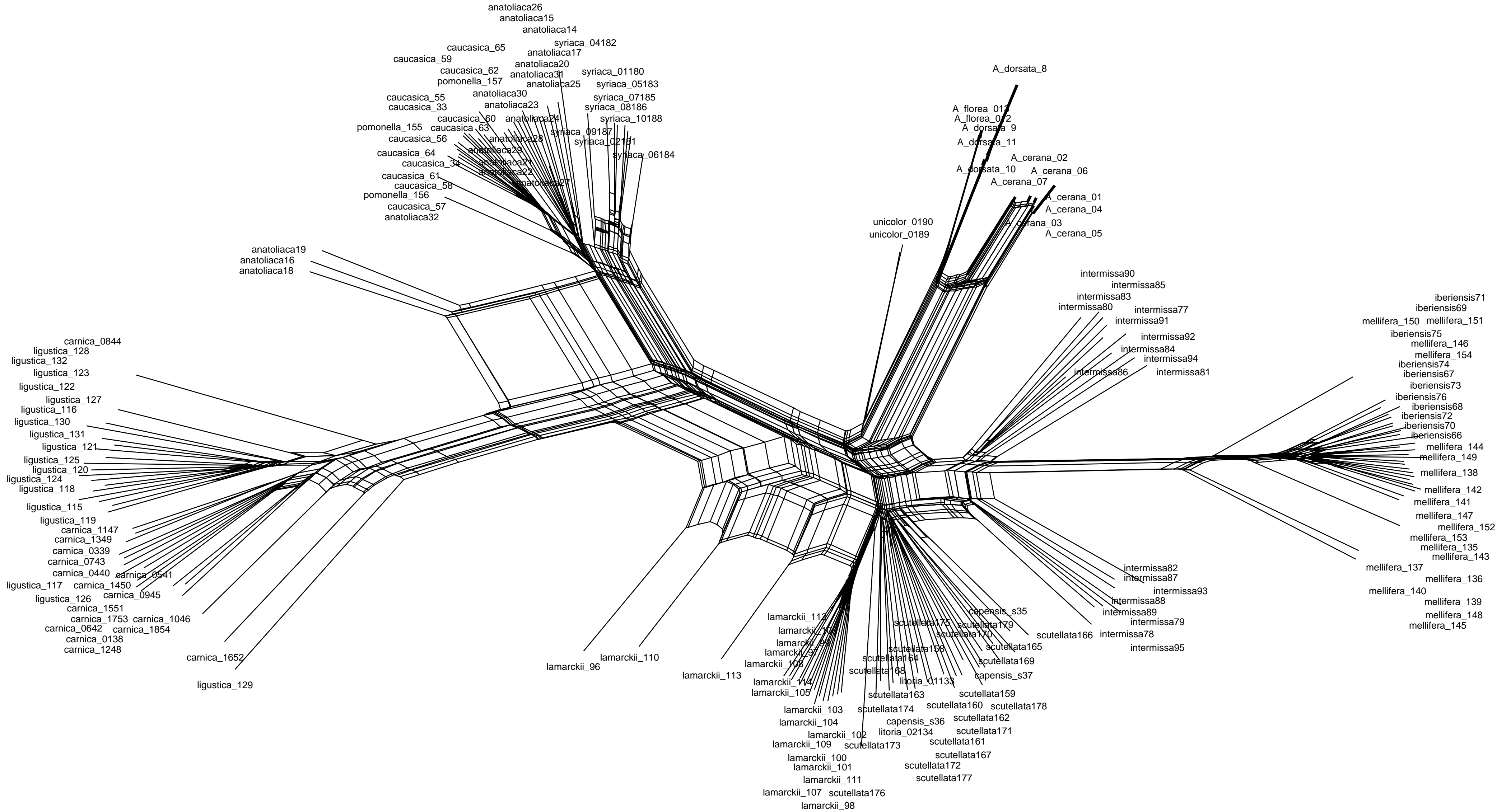

Supplement: Supplementary file 1 [file ece30002-1949-SD1.pdf]

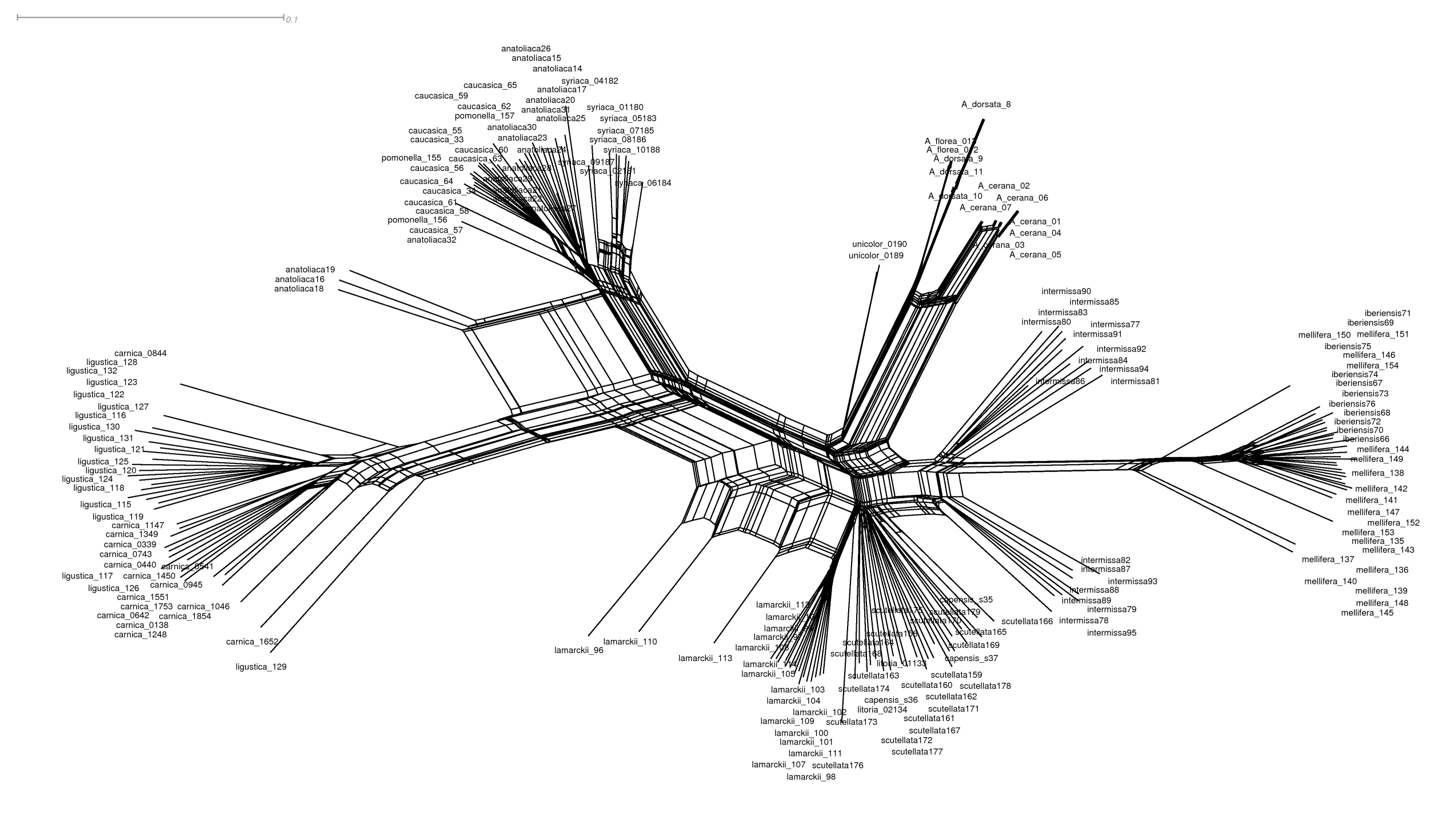

Supplement: Supplementary file 2 [file ece30002-1949-SD3.png]

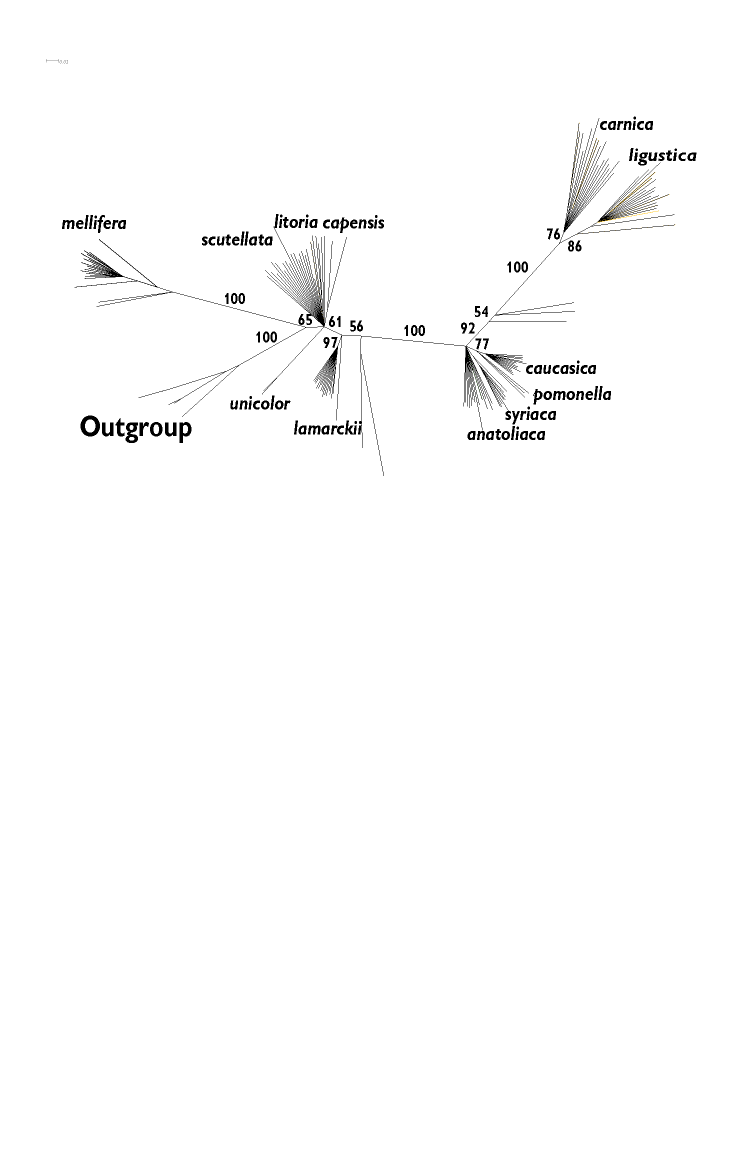

Supplement: Supplementary file 5 [file ece30002-1949-SD4.png]
