# Supplementary material for: From where did the Western honeybee (Apis mellifera) originate?
Source: Ecol Evol. 2012 Jul 12;2(8):1949–57. doi: 10.1002/ece3.312 (PMC3433997; doi:10.1002/ece3.312)
Supplement: Supplementary file 4 [file ece30002-1949-SD2.pdf]

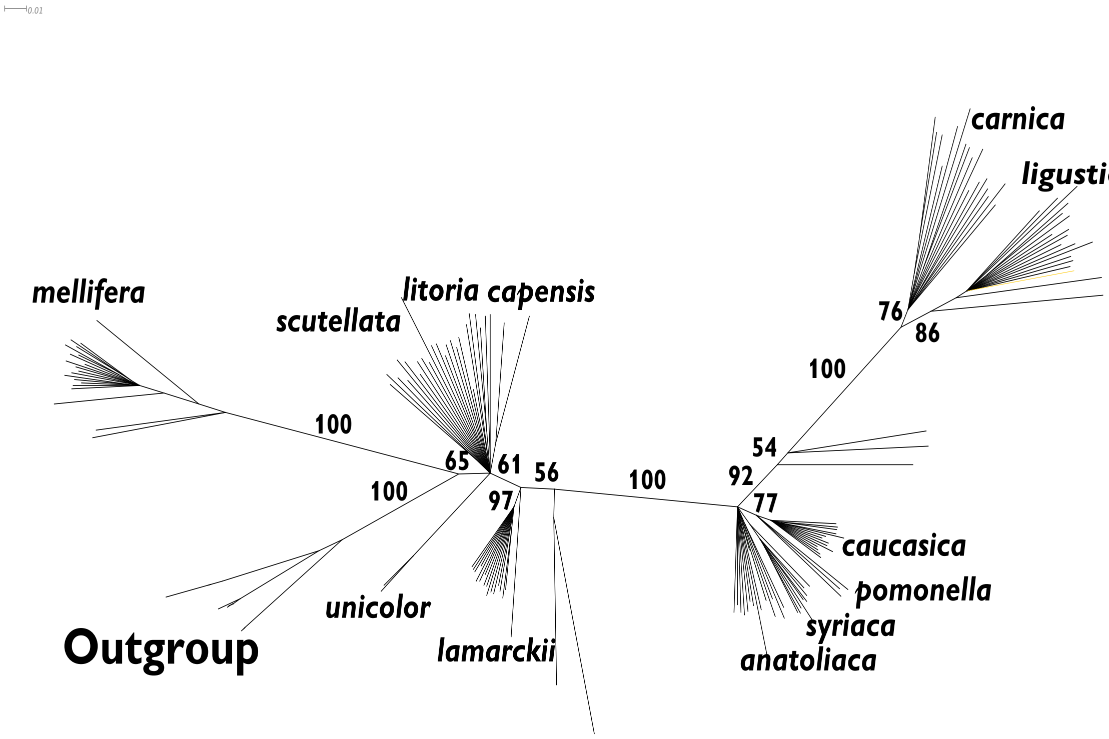
0.01
carnica
ligustica
76
86
100
mellifera
litoria capensis
scutellata
100
54
65
61
56
100
92
100
97
77
caucasica
pomonella
unicolor
syriaca
Outgroup
lamarckii
anatoliaca
